# The Avon Longitudinal Study of Parents and Children - A resource for COVID-19 research: Questionnaire data capture November 2020 – March 2021

**DOI:** 10.12688/wellcomeopenres.16950.2

**Published:** 2021-11-29

**Authors:** Daniel Smith, Claire Bowring, Nicholas Wells, Michael Crawford, Nicholas John Timpson, Kate Northstone

**Affiliations:** 1 ALSPAC, Department of Population Health Sciences, Bristol Medical School, University of Bristol, Bristol, BS8 2BN, UK; 2 MRC Integrative Epidemiology Unit, University of Bristol, Bristol, UK

**Keywords:** ALSPAC, Children of the 90s, birth cohort study, COVID-19, coronavirus, online questionnaire, mental health

## Abstract

The Avon Longitudinal Study of Parents and Children (ALSPAC) is a prospective population-based cohort study which recruited pregnant women in 1990-1992 and has followed these women, their partners (Generation 0; G0) and their offspring (Generation 1; G1) ever since. The study has reacted rapidly and repeatedly to the coronavirus disease 2019 (COVID-19) pandemic, deploying online questionnaires throughout the pandemic. In November/December 2020, a fourth questionnaire was deployed asking about physical and mental health, lifestyle and behaviours, employment and finances.

G0 participants were offered an online questionnaire between 17 ^th^ November 2020 and 7 ^th^ February 2021, while G1 participants were offered both online and paper questionnaires between 1 ^st^ December 2020 and 19 ^th^ March 2021. Of 15,844 invitations, 8,643 (55%) participants returned the questionnaire (3,101 original mothers [mean age 58.6 years], 1,172 original fathers/partners [mean age 61.5 years] and 4,370 offspring [mean age 28.4 years]). Of these 8,643 participants, 2,012 (23%) had not returned a previous COVID-19 questionnaire, while 3,575 (41%) had returned all three previous questionnaires.

In this questionnaire, 300 participants (3.5%) reported a previous positive COVID-19 test, 110 (1.3%) had been told by a doctor they likely had COVID-19, and 759 (8.8%) suspected that they had had COVID-19. Based on self-reported symptoms, between October 2020 and February 2021 359 participants (4.2%) were predicted COVID-19 cases.

COVID data is being complemented with linkage to health records and Public Health England pillar testing results as they become available. Data has been released as an update to the previous COVID-19 datasets. It comprises: 1) a standard dataset containing *all* participant responses to both questionnaires with key sociodemographic factors; and 2) as a composite release coordinating data from the existing resource, thus enabling bespoke research across all areas supported by the study. This data note describes the fourth questionnaire and the data obtained from it.

## Introduction

At the time of writing (May 2021), we are over a year into the coronavirus disease 2019 (COVID-19) pandemic. The global impact has been considerable, with over 165 million confirmed cases – and over three million deaths – to date ( WHO COVID-19 Dashboard). Despite the roll-out of vaccines, many countries are still under some form of restrictions, including local or national lockdowns. In the UK, mitigation strategies against the COVID-19 pandemic have changed repeatedly since March 2020, from an initial comprehensive set of restrictions on normal life (termed the ‘lockdown’) which took effect on March 23 ^rd^ 2020, to easing throughout summer 2020, followed by more targeted ‘local’ restrictions in Autumn 2020, back to second (in November 2020) and third (from early January 2021) nationwide lockdowns (with differing rules in different UK nations). More detailed information on the COVID timeline in the UK can be viewed here. Since 29 ^th^ March 2021, a gradual lifting of the restrictions has been introduced in the UK, such as a return to school and groups of up to six people being allowed to meet outdoors (whilst adhering to social distancing).

The impact of the COVID-19 pandemic and its management, including continuing restrictions, has lasted over a year; the long-term impact of COVID-19 on current and future employment, physical and mental health, financial activity and personal relationships is therefore likely to be substantial. It is vital that longitudinal, population-based studies continue to prospectively measure the impact of lockdowns and various management strategies on their participants. Such studies are needed to understand the ongoing effects of mitigation strategies on health and well-being ^1^ and identifying inequalities in response to the pandemic ^2^.

The Avon Longitudinal Study of Parents and Children (ALSPAC) is a unique three-generational study, comprising ‘G0’: the cohort of original pregnant women and the fathers/partners of these women; ‘G1’: the cohort of index children; and ‘G2’: the cohort of offspring of the index children. The study has a wealth of biological, genetic and phenotypic data across these generations ^3– 6^. ALSPAC has been well placed to capture information across key parts of the population during the COVID-19 pandemic – in particular the contrast between those in higher risk (the G0 cohort; mean age ~59 years) and lower risk (the G1 cohort; mean age ~28 years) groups. We have been able to collect repeat data quickly using our existing infrastructure for online data collection. So far, ALSPAC has conducted three COVID-19 questionnaires using G0 and G1 data: the first between 9 ^th^ April to 15 ^th^ May 2020 ^7^, the second between 26 ^th^ May to 5 ^th^ July 2020 ^8^; and the third, with an antibody test, between 3 ^rd^ and 20 ^th^ October 2020 ^9^. As part of the second questionnaire, parents of G2 children also completed a questionnaire for each of their children ^10^.

The wider COVID-19 data collection in ALSPAC will include data from three main sources: self-reported data from questionnaires, data from clinical services based on linkage to medical and other records (such as Public Health England Pillar testing ^11^) and information from biological samples. The data from these sources are intended to be complementary and help address different potential research questions around COVID-19.

This data note describes the data collected via our *fourth* questionnaire between 17 ^th^ November 2020 and 19 ^th^ March 2021 and provides a summary of the participants who responded.

The update to the datasets obtained from our previous questionnaires ^7– 9^ is described here, together with any variables that have been derived using multiple sets of questionnaire data. We also present a brief assessment of the factors associated with returning the fourth COVID questionnaire, but not any of the previous three, in order to explore who these ‘new’ participants are.

## Methods

### Setting

ALSPAC is an intergenerational longitudinal cohort that recruited pregnant women residing in Avon, UK with expected dates of delivery 1 ^st^ April 1991 to 31 ^st^ December 1992 ^3, 4^. The initial cohort consisted of 14,541 pregnancies resulting in 14,062 live births and 13,988 children who were alive at one year of age. From the age of seven onwards, the initial sample was bolstered with eligible cases who had originally failed to join the study and there were subsequently 14,901 children alive at one year of age following this further recruitment ^5^. Please note, the study website contains details of all the data that is available through a fully searchable data dictionary and variable search tool.

In response to the COVID-19 pandemic it was necessary to develop a data collection strategy which was practical, would yield data quickly and could be updated and repeated if necessary. For these reasons, we initially chose to use an online-only data collection approach for this, restricting our invites to those participants with a valid email address (and coordinated with a systematic communications/outreach campaign to obtain updated information from participants). For the fourth COVID-19 questionnaire, G0 data collection was again online-only. However, for G1 participants this COVID questionnaire was embedded within the wider ‘Life@28’ questionnaire as part of ALSPAC’s annual questionnaire strategy; this questionnaire was therefore available to G1 participants in both online and paper formats. This meant that, unlike previous questionnaires, G1 participants without a valid email address were sent a physical invitation letter through the post. For G0 participants, invites were only sent to those with a valid email address. Therefore, due to these differing data collection methods, unlike previous COVID-19 questionnaires where G0 and G1 data collection started and ended on the same dates, for this fourth COVID questionnaire start and end dates differed slightly between G0 and G1 participants (as detailed below in the 'Invitation and reminder strategy' section).

The online questionnaire was developed and deployed using Research Electronic Data Capture (REDCap) tools ^12^); a secure web application for building and managing online data collection exercises, hosted at the University of Bristol. Paper questionnaires were designed, scanned and verified using Teleform data capture software (version 16.5 (19) 22/10/2018). As this COVID-19 questionnaire was part of the usual ALSPAC annual data collection strategy for G1 we did not collect data from parents of G2 children not enrolled as G1 offspring (i.e., G1 partners).

### Content design

Content for our fourth questionnaire was selected to address three needs:

1. The need to track changes in health and wellbeing over time using repeated measures. To address this, we repeated a panel of questions from our previous questionnaires (e.g., health symptoms, recent contacts, mental health). By repeating questions, we are also able to capture information about participants who did not complete the previous COVID questionnaires.
2. The need to harmonize data collection with other cohorts to facilitate co-ordinated analyses as part of the National Core Studies Longitudinal Health and Wellbeing work programme. We addressed this by incorporating content from a coordinated and freely available COVID-19 questionnaire co-developed by ALSPAC. This questionnaire was developed in consultation with a network of UK and international longitudinal population studies and partners through a process facilitated by Wellcome.
3. The need to gather data to investigate specific hypotheses which could not be tested using the previous questionnaires. These topics were suggested by our collaborators and are detailed below.

The questionnaire included six sections, and captured information on the following (if the questions come from a standardised questionnaire, the source and reference has been provided in brackets; questions asked repeated from previous COVID-19 questionnaires have been noted in brackets as well):

1. Ethnicity
  • Information on ethnicity, based on categories used by the Office for National Statistics (note that this was part of the standard ‘Life@28’ annual ALSPAC questionnaire for G1 participants, not part of the COVID sections, but has been included here for comparison with the G0 generation)
2. Health
  • Symptoms of COVID-19 and negative control symptoms since October 2020 (symptoms repeated from Q1, Q2 and Q3)
  • Diagnosis with COVID-19 (repeated from Q1, Q2 and Q3) and details of symptoms (if infected)
  • Other health-related issues (restricting physical activities, shortness of breath) and further questions about long COVID, including cognitive/concentration issues
3. Your lifestyle
  • Eating habits before and during lockdown (some questions repeated from Q2)
4. Impact of the pandemic on feelings
  • Depression assessed using the Short Mood and Feelings Questionnaire (SMFQ; ^13^; repeated from Q1 and Q2)
  • Anxiety assessed using the General Anxiety Disorder-7 questionnaire (GAD7; ^14^; repeated from Q1 and Q2)
  • Well-being assessed using the Warwick-Edinburgh Mental Wellbeing Scales (WEMWBS; ^15^; repeated from Q1 and Q2)
  • Perception of risk (repeated from Q2)
  • Coping with life
  • Obsessive compulsive symptoms assessed using five items from the Obsessive-Compulsive Inventory-Revised ^16^ (of the original 18 items in this scale, five were chosen that loaded highest in the factor analysis of the original measure)
  • Reactions to stressful situations assessed using the Perceived Stress Scale ^17^
  • Whether key life events occurred since March 2020, and affect this had on participant
  • Free text inviting participants to provide details of other ways they have been affected by the pandemic
5. Healthcare use
  • Whether medical treatments or appointments have been cancelled or postponed during the COVID-19 pandemic, who cancelled them, and how worried participants were about this
  • Whether participant has developed signs or symptoms during the pandemic, whether they contacted a GP or healthcare professional, and how worried participants were about this
  • Thoughts about getting COVID-19 vaccine, and reasons for wanting or not wanting a vaccine
  • Effect of pandemic on plans to have children (G1 only; repeated from Q1)
6. Living, working and earning
  • Current living arrangements (repeated from Q2)
  • Whether living arrangements have changed since July 2020 (repeated from Q2)
  • Current employment circumstances (repeated from Q2)
  • Days spent working from home in past week and before COVID-19
  • Managing financially
  • Social contacts and methods of communication (repeated from Q1 and Q2)

The final questionnaire (REDCap PDF) used is available with the associated data dictionary (which includes frequencies of all variables that are available) as *Extended data* ^18^.

### Invitation and reminder strategy

Unlike previous ALSPAC COVID questionnaires, the invitation and reminder strategies differed for G0 and G1 cohorts, so will be explained here in turn. In all cases, participants were not contacted if our administrative database record indicated that they were deceased, had withdrawn from the study, had declined further contact or had declined to complete questionnaires.

On the 26 ^th^ and 27 ^th^ November 2020, all G0 participants for whom we had an active email address were sent an invitation to complete the questionnaire (n=6,721; note that this includes 12 G0 mother participants sent a questionnaire after this date as a result of outreach work undertaken by the ALSPAC team). On the 10 ^th^ December 2020 and 6 ^th^ January 2021, any non-responders were sent a reminder email or text respectively to complete the questionnaire, and a final text message was sent on 22 ^nd^ January 2021 to those for whom we had a current mobile phone number. The questionnaire survey was live on the online platform for nearly three months, with G0 questionnaire data collection turned off on 7 ^th^ February 2021.

All G1 participants with an active email address or known home address, and who had not withdrawn from the study, were sent an invitation to complete the online questionnaire on 2 ^nd^ December 2020 (n=9,123; including five G1 participants sent a questionnaire after this date as a result of outreach work undertaken by the ALSPAC team). On the 15 ^th^ December 2020 and 5 ^th^ January 2021, any non-responders were sent a reminder email to complete the online questionnaire. As part of our general questionnaire strategy, and in an effort to reduce paper printing, we elected to only send paper-based questionnaires to those participants who were deemed more likely to complete on paper. We defined this as participants who had completed at least one of the last three annual questionnaires on paper. Just over 1,000 paper questionnaires were sent out on 6 ^th^ January 2021. A letter was sent between 18 ^th^ and 22 ^nd^ January and finally a text reminder on the 29 ^th^ January to non-responders. The questionnaire was live on the online platform for nearly three months, with G1 questionnaire data collection turned off on 15 ^th^ February 2021. ALSPAC stopped counting paper questionnaires on 19 ^th^ March 2021.

In addition, traditional (print, radio, television) and social media (Facebook, Instagram and Twitter) were used to inform participants that the questionnaire was live, asking them to contact us if they had not received it and to encourage completion. These communication channels were also used to encourage re-engagement of friends and family back into the study. Unlike our standard questionnaires (usually completed annually), we did not provide any incentive for G0 completion. As the fourth COVID questionnaire was embedded within the wider annual questionnaire, G1 participants were offered an incentive (a £10 shopping voucher) for returning a questionnaire. For both G0 and G1, we offered a prize draw (three prizes of £100) for those who completed their questionnaire by 31 ^st^ January 2021 (for G0 participants) or 7 ^th^ February 2021 (for G1 participants).

### Response rate

A total of 15,844 invitations were sent out and responses were received from 8,643 participants (overall response rate of 55%).

As with our previous COVID questionnaires, female G1 participants were much more likely to respond than male G1 participants. Table 1 summarises the response rate within each group organised by cohort structure. Response rate amongst G0s was higher for the fourth questionnaire (64%; 4,273/6,721) compared to the first (57%; 3,720/6,393) ^7^ and second (58%; 3,678/6,467) ^8^. Response rate for the G1 cohort (48%; 4,370/9,123) was slightly higher than for the second COVID-19 questionnaire (44%; 2,711/6,148) ^8^, but lower than response to the first questionnaire (51%; 2,973/5,842) ^7^. At face value, the £10 gift voucher incentive for G1s did not appear to increase response rates for this questionnaire. However, this may be explained by the fact that earlier COVID-19 questionnaires only went out to those with valid email addresses, whereas this questionnaire was also sent to G1 participants via post, meaning this fourth COVID-19 questionnaire was sent to a larger sample of G1 participants. These ‘new’ participants may be less engaged with the study, and hence less likely to return a questionnaire, which may balance out the benefit of having an incentive. Supporting this idea, if we split the G1 data according to whether they were sent an invitation for the second COVID-19 questionnaire, of the 6,143 participants who were sent both the second and fourth COVID-19 questionnaire invitations, 4,005 (65%) returned the fourth COVID-19 questionnaire. In contrast, of the 2,980 participants who were not invited to complete the second COVID-19 questionnaire but were invited to complete the fourth COVID-19 questionnaire, only 365 (12%) returned one.

**Table 1.** Number of participants who were eligible and who responded to the fourth COVID-19 questionnaire (Q4).

Cohort Group	Eligible ^1^	Responded to Q4 ^2^
G0 Mothers	4852	3101 (64%)
G0 Fathers/partners	1869	1172 (63%)
G1 Offspring daughters	4925	2883 (59%)
G1 Offspring sons	4198	1487 (35%)
**TOTAL**	**15844**	**8643 (55%)**

^1^G0 eligibility criteria (online questionnaires only): valid email address, marked as contactable for questionnaires; G1 eligibility criteria (online and paper questionnaires): marked as contactable for questionnaires.

^2^ Proportions of those invited (i.e. eligible).

Of the 8,643 respondents, 2,012 (23%) had not returned a previous COVID questionnaire, 3,575 (41%) had returned all three of the previous COVID questionnaires, with 3,056 (35%) returning one or two (but not all three) of the previous COVID questionnaires ( Table 2). Of those who returned a fourth COVID questionnaire, G1 participants were more likely to have not completed a previous COVID questionnaire (31% of G1s, vs 16% of G0 mothers and 15% of G0 fathers/partners).

**Table 2.** Number of participants who responded to the fourth COVID-19 questionnaire and whether they completed previous ALSPAC COVID-19 questionnaires.

Previous COVID-19 questionnaires	G0 mothers	G0 fathers/ partners	G1 offspring	Total
No previous COVID-19 data	489 (16%)	174 (15%)	1349 (31%)	2012 (23%)
Returned one or two (but not all three) previous COVID-19 questionnaires	1564 (50%)	562 (48%)	1449 (33%)	3575 (41%)
Returned all three previous COVID-19 questionnaires	1048 (34%)	436 (37%)	1572 (36%)	3056 (35%)
**TOTAL**	**3101**	**1172**	**4370**	**8643**

### Key results

Characteristics of responders according to key variables that will be released with the complete dataset can be seen in Table 3. The population who responded were predominantly white (> 96%) and the majority had at least A-level qualifications (optional exams in the UK sat at the age of 18 years), with 54% of G0 mothers, 70% of G0 partners/fathers and 74% of G1 offspring in this category. G0 partners/fathers were three years older on average than G0 mothers (61.5 years vs 58.6 years), with G1 offspring having an average age of 28.4 years.

**Table 3.** Summary of key characteristics for those who responded to the fourth COVID questionnaire; n (%) for categorical variables or mean (sd) for continuous variables. The sample size for each characteristic is given in brackets after the % (for categorical variables) or sd (for continuous variables). Total sample sizes are 3,101 for G0 mothers, 1,172 for G0 fathers/partners, and 4,370 for G1 offspring.

Key characteristic	G0 Mothers	G1 Fathers/ partners	G1 Offspring
Age (years)	58.6 (4.4; *n* = 3,101)	61.5 (5.15; *n* = 1,172)	28.4 (0.53; *n* = 4,366 ^4^)
Latest BMI ^1^	26.4 (5.11; *n* = 2,459)	27.46 (4.04; *n* = 896)	24.6 (5.15; *n* = 3,553)
Latest systolic BP ^1^	119.6 (14.3; *n* = 2,449)	132.8 (13.21; *n* = 904)	116 (11.28; *n* = 3,485)
Latest diastolic BP ^1^	70.8 (9.39; *n* = 2,449)	77.4 (9.02; *n* = 904)	66.6 (7.85; *n* = 3,485)
Education level ^2^ ≥A level	1593 (53.8%; *n* = 2,962)	776 (69.8%; *n* = 1,112)	2296 (73.6%; *n* = 3,118)
Ethnicity (from baseline ALSPAC) ^3^ White	2900 (98.2%; *n* = 2,953)	1098 (98.7%; *n* = 1,112)	3740 (96.3%; *n* = 3,885)
Ethnicity (from COVID questionnaire) White	3033 (98.0 %; *n* = 3,096)	1150 (98.3%; *n* = 1,170)	4182 (95.8%; *n* = 4,362)

^1^Data taken from the most recent clinic that individual attended where available.

^2^Data taken from pregnancy questionnaires for G0 and from most recent questionnaire for G1 where available.

^3^Data taken from pregnancy questionnaires for all.

^4^This sample size is lower than the total number of G1’s who returned a questionnaire ( *n* = 4,370) as data for four triplet/quadruplet pregnancies have been coded as missing in the release dataset for confidentiality reasons.

BMI, body mass index; BP, blood pressure; sd, standard deviation.

As with the previous questionnaires, participants were asked whether they thought they have had COVID-19. Options were: ‘Yes, confirmed by a positive test’, ‘Yes, suspected by a doctor but not tested’, ‘Yes, my own suspicions’ or ‘No’. In the fourth questionnaire, 300 (3.5%) respondents reported that they had tested positive to COVID-19, 110 (1.3%) reported that COVID-19 was suspected by a doctor but not tested and 759 (8.8%) believed they had COVID-19 due to their own suspicions. Table 4 summarises the responses to this question by cohort structure.

**Table 4.** Participant response to whether they have had COVID-19 from the fourth COVID-19 questionnaire.

	G0 mothers	G0 partners/ fathers	G1 offspring	Total
Yes, positive test	69 (2.3%)	26 (2.2%)	205 (4.7%)	300 (3.5%)
Yes, doctor suspected, no test	33 (1.1%)	10 (0.9%)	67 (1.5%)	110 (1.3%)
Yes, own suspicions	205 (6.7%)	97 (8.4%)	457 (10.5%)	759 (8.8%)
No	2757 (90%)	1026 (88.5%)	3631 (83.3%)	7414 (86.4%)

As with the previous questionnaires, we applied the algorithm derived by Menni and colleagues ^19^ to predict ‘probable infection’ using data collected from an app-based symptom tracker ^20^. This algorithm had sensitivity of 0.65 and specificity of 0.78 ^19^. This algorithm uses four symptoms: loss of smell and taste, severe or significant persistent cough, severe fatigue and skipped meals (coded as 1 if present and 0 otherwise), together with age and sex (1 male; 0 female). We had slight differences in wording and thus the algorithm (using the same weightings) applied was as follows:

-1.32 - (0.01 x age) + (0.44 x sex) + (1.75 x loss of loss of smell *or* taste)
+ (0.31 x *new* persistent cough) + (0.49 x severe fatigue)
+ (0.39 x decreased appetite).

**Figure 1.**
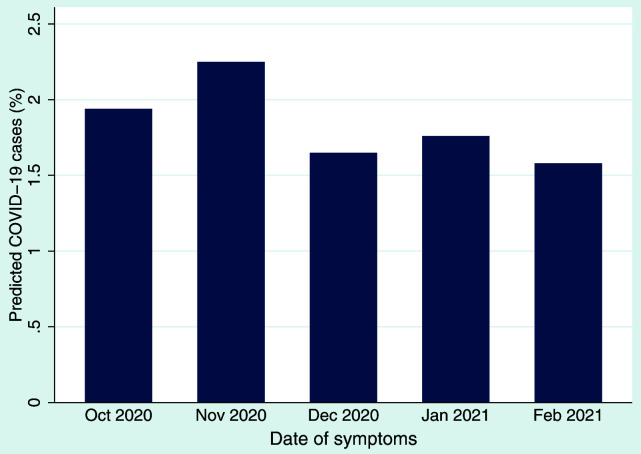
Predicted cases (% of population) of COVID-19 per month according to symptoms reported for those months using the Menni *et al.* algorithm ^19^.

Probable COVID-19 cases were obtained by applying an exp( *x*)/[1+(exp( *x*)] transformation and coding values >0.5 as probable cases. We applied this algorithm to our monthly symptom data collected in questionnaire 4 which asked about symptoms experienced from October 2020 until February 2021. The proportion of predicted cases decreased slightly over time, with 1.9% and 2.3% of predicted cases in October and November 2020, respectively, and 1.6–1.8% of predicted cases in December 2020 to February 2021 ( Figure 1). These monthly percentages are lower than the peak in March 2020 (4.2%) observed from our first COVID-19 questionnaires ^7^. Combining all of the questionnaire 4 data together, 359 of 8,587 (4.2%) of participants were predicted to be COVID-19 cases between October 2020 and February 2021. As with our previous use of this algorithm, we note that these predictions are subject to important assumptions, which we discussed in more detail in our previous data notes ^7, 8^: 1) the baseline risk of having COVID-19 (intercept term) is the same as in the Menni study population; 2) the slight difference in the wording of symptoms captures the same information as those in the Menni study; and 3) the association of these symptoms with COVID-19 (fixed-effect terms) are the same as in the Menni study population.

As these data have been collected repeatedly, we can combine these results across each of the COVID questionnaires. For self-reported COVID-19 status, of those who completed this question for any of the four COVID questionnaires, 499 participants (5.1%) have either had a positive test or a doctor suspected that they had had COVID-19. G1s were more likely to report having had a positive COVID-19 test or doctor-suspected symptoms, compared to the G0 generation ( Table 5). If we include ‘own suspicions’ in our case definition of COVID-19 infection, the number of participants who may have had COVID-19 increases to 2,231 (23.0%). Similar analyses based on predicted COVID-19 cases from the Menni algorithm from March 2020 onwards show that 961 (9.9%) were predicted cases. These results are broadly similar – albeit slightly lower – if we restrict our analysis to those who responded to all four COVID questionnaires; by doing so we remove any potential bias due to: i) differences in COVID-19 testing, since widespread testing was not available during the time of the first COVID-19 questionnaire; and ii) the time available to be infected, which could bias results if only including those who responded to the early COVID questionnaires ( Table 5).

**Table 5.** Self-report and predicted COVID-19 cases across all four COVID questionnaires.

	Sample	G0 mothers	G0 partners/ fathers	G1 offspring	Total
Self-reported positive test OR doctor suspected	Answered *any* COVID Qs	135 (3.84%)	45 (3.33%)	319 (6.60%)	499 (5.14%)
	Answered *all* four COVID Qs	58 (4.06%)	17 (2.84%)	114 (5.77%)	189 (4.72%)
Self-reported positive test OR doctor suspected OR own suspicions	Answered *any* COVID Qs	719 (20.43%)	258 (19.08%)	1254 (25.94%)	2231 (22.98%)
	Answered *all* four COVID Qs	260 (18.22%)	82 (13.69%)	499 (25.27%)	841 (21.02%)
Predicted cases based on symptoms (from March 2020 onwards)	Answered *any* COVID Qs	256 (7.27%)	107 (7.91%)	598 (12.37%)	961 (9.90%)
	Answered *all* four COVID Qs	80 (5.60%)	39 (6.51%)	235 (11.89%)	354 (8.84%)

**Figure 2.**
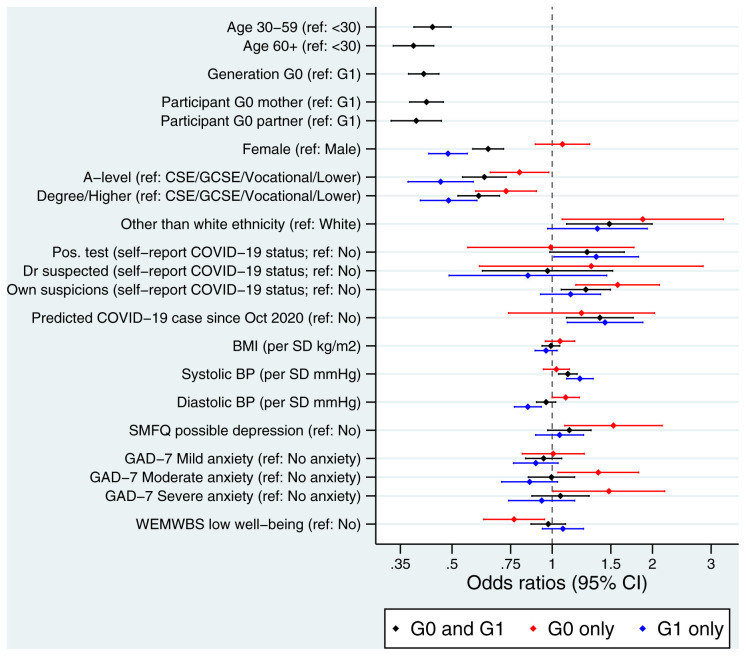
Forest plot describing the factors predicting returning the fourth COVID questionnaire, if none of the previous questionnaires were returned. All results are odds ratios from logistic regression models with ‘only returned questionnaire 4’ as the outcome. Results are displayed for the G0 and G1 participants combined (black; n = 8,643; n ‘only returned COVID questionnaire 4’ = 2,012; n ‘returned questionnaire 4 and at least one other previous COVID questionnaire’ = 6,631), the G0 participants separately (red; n = 4,273; n ‘only returned COVID questionnaire 4’ = 663; n ‘returned questionnaire 4 and at least one other previous COVID questionnaire’ = 3,610), and the G1 participants separately (blue; n = 4,370; n ‘only returned COVID questionnaire 4’ = 1,349; n ‘returned questionnaire 4 and at least one other previous COVID questionnaire’ = 3,021). Other than ‘age’, ‘generation’ and ‘participant’ (which are univariable models), all models for the G0 and G1 participants combined adjusted for ‘generation’ (G0 vs G1). All G0-only and G1-only models are univariable models. Results to the right of the dashed line indicate an increased odds of only returning questionnaire 4 relative to the reference category, while results to the left indicate a decreased odds. The x-axis is on a logarithmic scale. Sample sizes and percentage of missing data: Age (n G0/G1 = 8,639; % missing = 0.05%); Generation (n G0/G1 = 8,643; % missing = 0.0%); Participant cohort (n G0/G1 = 8,643; % missing = 0.0%); Sex (n G0/G1 = 8,643; % missing = 0.0%: n G0-only = 4,273; % missing = 0.0%: n G1-only 4,370; % missing = 0.0%); Education (n G0/G1 = 7,190; % missing = 16.8%: n G0-only = 4,074; % missing = 4.7%: n G1-only 3,116; % missing = 28.7%); Ethnicity (n G0/G1 = 7,950; % missing = 8.0%: n G0-only = 4,065; % missing = 4.9%: n G1-only 3,885; % missing = 11.1%); Self-reported COVID-19 status (n G0/G1 = 8,583; % missing = 0.7%: n G0-only = 4,223; % missing = 1.2%: n G1-only 4,361; % missing = 0.2%); Predicted COVID-19 case from symptoms (n G0/G1 = 8,587; % missing = 0.7%: n G0-only = 4,226; % missing = 1.1%: n G1-only 4,361; % missing = 0.2%); BMI (n G0/G1 = 6,903; % missing = 20.1%: n G0-only = 3,355; % missing = 21.5%: n G1-only 3,548; % missing = 18.8%); Systolic blood pressure (n G0/G1 = 6,835; % missing = 20.9%: n G0-only = 3,353; % missing = 21.5%: n G1-only 3,482; % missing = 18.8%); Diastolic blood pressure (n G0/G1 = 6,835; % missing = 20.9%: n G0-only = 3,353; % missing = 21.5%: n G1-only 3,482; % missing = 18.8%); SMFQ (n G0/G1 = 8,236; % missing = 4.7%: n G0-only = 4,020; % missing = 5.9%: n G1-only 4,216; % missing = 3.5%); GAD-7 (n G0/G1 = 8,330; % missing = 3.6%: n G0-only = 4,083; % missing = 4.4%: n G1-only 4,247; % missing = 2.8%); WEMWBS well-being (n G0/G1 = 8,260; % missing = 4.4%: n G0-only = 4,121; % missing = 5.5%: n G1-only 4,223; % missing = 3.4%). BMI, body mass index; BP, blood pressure; CI, confidence interval; CSE, Certificate of Secondary Education; GAD-7; General Anxiety Disorder-7 questionnaire; GCSE, General Certificate of Education; SMFQ, Short Moods and Feelings Questionnaire; SD, standard deviation; WEMWBS, Warwick-Edinburgh Mental Wellbeing Scales.

To assess potential reasons for non-completion of the fourth COVID questionnaire, which could bias comparisons between questionnaire waves, we explored whether various sociodemographic factors were associated with returning the fourth COVID questionnaire, but not any of the previous three (to explore who these ‘new’ participants were). Results are displayed in Figure 2 for the G0/G1 participants combined (black), and for the G0 participants (red) and G1 participants (blue) separately. Returning only the fourth questionnaire was strongly associated with age/generation such that younger/G1 participants were much more likely to have only returned Q4 (most likely a result of the different G1 data collection strategy, which included mailing paper invitations to participants, and hence invited additional G1 participants not invited to the previous COVID-19 questionnaires). After adjusting for generation (G0 vs G1), response to only the fourth COVID questionnaire was associated with several factors: male participants, those with lower educational attainment (a proxy for socioeconomic position), and participants with an ethnicity other than white were more likely to only have fourth COVID questionnaire data. Participants who only returned the fourth COVID questionnaire were also more likely to self-report that they either had a positive COVID-19 test or had their own suspicions they had had COVID-19, relative to a reference group of ‘not had COVID-19’ (no difference for ‘doctor suspected COVID-19 infection’ was reported). These participants were also more likely to be a predicted COVID-19 case, based on their reported symptoms, using the Menni algorithm ^19^. Few differences in physical or mental health were noted, although participants who only completed the fourth COVID questionnaire had higher systolic blood pressure, and a somewhat elevated risk of potential depression. Some differences between the generations were noted, such as male participants and those with fewer educational qualifications being more likely to return only the fourth questionnaire amongst G1s, relative to G0s. G0s with more depressive symptoms, higher levels of anxiety and lower well-being were associated with being more likely to only return the fourth questionnaire, while this pattern was not observed in G1 participants.

## Strengths and limitations of the data

This data collection has a number of strengths. Firstly, the timelines within which the collection occurred allows comparisons to be made between the stringent mitigation measures early on in the pandemic and later easing of lockdown measures; users should ensure they are aware of completion dates for this dataset, as England went into second national lockdown between 5 ^th^ November and 2 ^nd^ December 2020, and a third national lockdown again on January 6 ^th ^2021 (with local tiered restrictions between these dates).

Secondly, the availability of repeat data obtained over time throughout the pandemic, along with pre-pandemic baseline measures, allows assessment of longitudinal changes in health and wellbeing. For example, we have already been able to demonstrate the short-term impact the pandemic has had on mental health during the first lockdown in April–June 2020 ^1, 21^. This is the fourth ALSPAC COVID-19 questionnaire, meaning there is a wealth of longitudinal data to draw upon to explore the impact of the COVID-19 pandemic and its management on population health. These questionnaires are also supplemented by serology testing ^9^ (with further testing currently being conducted [as of April/May 2021]), linking to Public Health England Pillar testing records ^11^, and targeted data collections (such as a long COVID-specific questionnaire currently in development).

Thirdly, the alignment of measures with other UK studies provides potential for cross-cohort comparisons. This was achieved through the set of core questions developed by the Wellcome coordinated group and has already facilitated a co-ordinated analysis of mental health measures in ALSPAC and Generation Scotland ^1^. ALSPAC is currently working on several cross-cohort projects to better understand the impact of the COVID-19 pandemic, including long COVID ( NIHR funded CONVALESENCE study), cardiovascular health ( COVIDITY), and longer-term mental health outcomes ( COVID-19 National Core Studies [NCS]), among others.

Finally, we achieved an excellent response rate despite the lack of incentive in G0 and calling on our participants to take part in data collection for the fourth time in less than a year. As we have described previously, it should be noted that our online-only strategy will likely have affected response rates from our G0 mothers who historically have tended to use paper questionnaires more than other sub-groups when completing questionnaires and for whom we are least likely to hold a current email address. However, the pandemic has led to a number of participants reaching out and getting in touch to provide these details, and indeed to re-engage with the study having dropped out previously. In addition, members of the study team have been contacting participants to ensure we have up to date email addresses. Throughout the pandemic, ALSPAC has also conducted outreach work to encourage participation of ‘disengaged’ study participants.

A key limitation of this data collection is that, in some cases, the data recorded is potentially identifiable. As with previous COVID-19 questionnaires, we have gone through each individual variable and made decisions as to whether we need to combine categories. This has only been carried out where we believe the data provides a high risk of potential disclosure (as detailed in the supplementary documentation file). Another limitation is that the response rate was non-random with regard to age, sex and socio-economic status; this could potentially introduce bias into analyses ^22^. We acknowledge there is a risk for people with severe COVID-19 to be under-represented in the study if they were too unwell to respond to questionnaires. The reverse may also be true, however; participants who have had COVID-19 (or believe that they have), may be more engaged with this research given their personal experience. For instance, in the first serology test (COVID questionnaire 3), participants who previously self-reported that they had had COVID-19 were more likely to participate in this research ^9^. We will be investigating this further using linkage to health records. Finally, we acknowledge that the predicted case status will contain measurement error. We will address this by providing more accurate measures of COVID-19 status in the future using a combination of serological testing (with a second serology test conducted in April 2020) ^23^ and data linkage ^11^.

A further potential limitation is that whilst data collection for G0 participants was comparable to previous ALSPAC COVID-19 questionnaires (i.e., email invitation to an online-only questionnaire with no incentive), for G1 participants the methodology for the fourth COVID-19 questionnaire differed slightly as it was part of our standard annual questionnaire process. This meant that: 1) both email and postal invitations were sent; 2) approximately 1,000 paper questionnaires were received; and 3) there was a £10 gift voucher incentive. This resulted in a larger G1 sample size for this COVID-19 questionnaire, which may impact our results, both between the G0 and G1 generations and for G1 participation compared to previous COVID-19 questionnaires. First, the G1 sample with questionnaire data is substantially larger than for previous COVID-19 questionnaires (4,370 at the fourth questionnaire, vs 2,973 and 2,711 at the first and second questionnaires, respectively). While larger sample sizes are positive and potentially allows us to fill in missing information in earlier questionnaires, G1 participants who only completed the fourth questionnaire will not have previous COVID-19 data from earlier in the pandemic which may limit their use in longitudinal analyses assessing the progressive impact of the pandemic on participants. Second, focusing just on G1 participants who were previously sent a second COVID-19 questionnaire, response rates were higher than for previous questionnaires (65% response vs 51% and 44% response rates in the first and second questionnaires, respectively), suggesting that the incentive enhanced response (additional details can be found in the ‘response rates’ section). Together, this different data collection strategy may have altered factors associated with response among G1 participants, compared to previous data collections. Focusing just on the G1-specifc results in Figure 2 (in blue), G1 participants who only completed the fourth COVID-19 questionnaire, relative to those who completed the fourth COVID-19 questionnaire plus at least one previous questionnaire, were more likely to be male and have fewer educational qualifications. These differences were less pronounced in the G0-specific findings (red results in Figure 2), suggesting that they may have been due to the different data collection methods employed for G1 participants. However, whether these differences are solely due to the different data collection methods, as opposed to other differences between the generations, is difficult to infer from these results. Nonetheless, researchers should be aware of these different data collection techniques when using these data.

In summary, this ALSPAC data obtained during the first year of the COVID-19 pandemic will enable researchers to capture changes in many aspects of people’s lives as we optimistically consider the pandemic abating and national mitigation strategies continuing to reduce. These data are available for researchers as described below.

## Consent

Completion of the questionnaire was optional and choosing to complete the questionnaire is considered informed consent for the questionnaire. 

Ethical approval for the study was obtained from the ALSPAC Ethics and Law Committee and the Local Research Ethics Committees. Informed consent for the use of data collected via questionnaires and clinics was obtained from participants following the recommendations of the ALSPAC Ethics and Law Committee at the time. Study participants have the right to withdraw their consent for elements of the study or from the study entirely at any time. Full details of the ALSPAC consent procedures are available on the study website.

## Data availability

### Underlying data

ALSPAC data access is through a system of managed open access. The steps below highlight how to apply for access to the data included in this data note and all other ALSPAC data:

1. Please read the ALSPAC access policy which describes the process of accessing the data and samples in detail, and outlines the costs associated with doing so.

2. You may also find it useful to browse our fully searchable research proposals database, which lists all research projects that have been approved since April 2011.

3. Please submit your research proposal for consideration by the ALSPAC Executive Committee. You will receive a response within 10 working days to advise you whether your proposal has been approved.

Please note that a standard COVID-19 dataset will be made available at no charge (see description below); however, costs for required paperwork and any bespoke datasets required additional variables will apply.

### Extended data

Open Science Framework: ALSPAC COVID-19 Data collections. https://doi.org/10.17605/OSF.IO/RM7HU ^18^.

This project contains the following extended data:

• ALSPAC_COVID_varlist.pdf (List of variable names and labels)

This project contains the following extended data within the folder ‘Questionnaire 4’:

• ALSPAC COVID Q4 FINAL (G0).pdf (REDCap PDF of final G0 questionnaire)
• ALSPAC COVID Q4 FINAL (G1).pdf (REDCap PDF of final G1 questionnaire)
• ALSPAC COVID Q4 data dictionary.pdf (Associated data dictionary including frequencies of all variables that are available)

Data are available under the terms of the Creative Commons Attribution 4.0 International license (CC-BY 4.0).

### COVID-19 Questionnaire 4 Data File

Data from the fourth ALSPAC COVID-19 questionnaire is available in two ways.

1. A freely available standard set of data containing *all* participants together with key sociodemographic variables (where available) is available on request (see data availability section). This dataset also includes data obtained from the previous COVID questionnaires. Subject to the relevant paperwork being completed (costs may apply to cover administration) this dataset will be made freely available to any bona fide researcher requesting it. Variable names will follow the format *covid4_xxxx* where *xxxx* is a four-digit number. A full list of variables released is available as *Extended data* ^18^. Frequencies of variable and details of any coding/editing decisions and derived variables are also available in the data dictionary as *Extended data* ^18^.
2. Formal release files have been created for G0 mothers, G0 fathers and G1 participants in the usual way and now form part of the ALSPAC resource. These datasets (or sections therein) can be requested in the usual way. Variable names will replicate those in 1) above but as each variable in ALSPAC is uniquely defined we have added markers to denote the source of the variable. For example, in the fourth COVID-19 questionnaire dataset, the age of the participant at completion (in years) is denoted by *covid4_9650*. In the G0 mother’s dataset this will be denoted by *covid4m_9650*, for G0 fathers/partner this will be *covid4p_9650* and for the G1 generation it will be *covid4yp_9650*. Frequencies for all variables for each participant group are available in the data dictionary in the usual way.

Text data and other potentially disclosive information will not be released until they have been coded appropriately. Table 6 describes the data that is withheld at the time of first release. Data will be incorporated back into both file sets as they become available.

**Table 6.** Data from questions that will not be released until coded.

Question number	Question text
**Section 1 - Ethnicity**	
B1_otherWhite	Details of other white background
B1_otherMixed	Details of other mixed/multiple ethnic background
B1_otherAsian	Details of other Asian background
B1_otherBlack	Details of other Black/African/Caribbean background
B1_other	Details of other ethnic group
**Section 2 – Health**	
2a	Date told or when first thought had COVID-19
2h	Details of new condition, illness or disability as a consequence of infection with coronavirus/COVID-19
**Section 4 – Impact on your feelings during the pandemic**	
9	Is there anything else you would like to tell us about how the pandemic has affected you?
**Section 5 – Healthcare use**	
1a	Details of other medical treatments of appointments cancelled or postponed
3a	Other reason don’t want to get vaccinated against COVID-19
3b	Other reason do want to get vaccinated against COVID-19
**Section 6 – Living, working and earning**	
1a_v	Details of other family member(s) live with
1a_viii	Details of other people live with
2a	Details of other change in living arrangements since July 2020
