# Supplementary material for: The Avon Longitudinal Study of Parents and Children - A resource for COVID-19 research: Questionnaire data capture November 2020 – March 2021
Source: Wellcome Open Res. 2021 Nov 29;6:155. Originally published 2021 Jun 16. [Version 2] doi: 10.12688/wellcomeopenres.16950.2 (PMC8591520; doi:10.12688/wellcomeopenres.16950.2)
Supplement: Peer review history

# Peer review: Reviewer response for version 1

**DOI:** 10.21956/wellcomeopenres.18704.r46523

**Published:** 2021-11-11

**Authors:** An Pan, Yi Wang

**Affiliations:** 1 Department of Epidemiology and Biostatistics, School of Public Health, Tongji Medical College, Huazhong University of Science and Technology, Wuhan, China; 2 Huazhong University of Science and Technology, Wuhan, China

This article presented a detailed introduction of the 4 ^th^ questionnaire on COVID-19 between Nov 17 ^th^ 2020 and March 19 ^th^ 2021 in the Avon Longitudinal Study of Parents and Children (ALSPAC), which is a large prospective population-based cohort study originally established in 1990-1992. The authors described the content and the implementation of the 4 ^th ^survey during COVID-19 pandemic, and also showed the response rate and the COVID-19 status according to different types of participants. This longitudinal study with repeated investigations may help to assess the impact of COVID-19, either from the disease per se or the public management measures, on the changes in many aspects for participants of different generations. This article has a very clear and comprehensive description of the study design.

 I have some minor inquiries and suggestions.

1. The authors collected data from G0 only via online questionnaire, whereas from G1 via both online and post in the 4 ^th ^survey, which was also different from the previous COVID-19 surveys. We noticed that the authors also have mentioned a larger sample of G1 participants in the 4 ^th ^survey may explain the change of the G1 response rate as compared to previous surveys. The authors should further discuss the potential influence of this issue.
2. Please provide references when comparing the response rates between different surveys. In addition, it was not quite clear what data were collected from the previous three surveys. Therefore, a table to summarize all variables or data in the four surveys might be helpful.
3. I am not quite convinced to use the variable “predicted COVID-19 cases”, particularly if this was the outcome variable. Sensitivity and specificity of the variable should be provided.
4. I am not sure whether the authors could use serological testing in the future to know the infection status, given that many of the participants could have obtained the vaccines. Should the vaccine information be included in the questionnaires?
5. Perhaps the authors could consider adding the major challenges and a future perspective in the context of COVID-19 pandemic.

Are sufficient details of methods and materials provided to allow replication by others?

Yes

Is the rationale for creating the dataset(s) clearly described?

Yes

Are the datasets clearly presented in a useable and accessible format?

Yes

Are the protocols appropriate and is the work technically sound?

Yes

Reviewer Expertise:

Chronic disease epidemiology

We confirm that we have read this submission and believe that we have an appropriate level of expertise to confirm that it is of an acceptable scientific standard.

# Response

**Published:** 2021-11-26

**Authors:** Daniel Smith

**Affiliations:** University of Bristol, UK

We thank the reviewers for their positive and constructive comments, and we hope that our revisions have addressed any remaining queries/suggestions. The reviewer’s comments are below, followed by our responses in italics.

**I have some minor inquiries and suggestions.**

**The authors collected data from G0 only via online questionnaire, whereas from G1 via both online and post in the 4th survey, which was also different from the previous COVID-19 surveys. We noticed that the authors also have mentioned a larger sample of G1 participants in the 4th survey may explain the change of the G1 response rate as compared to previous surveys. The authors should further discuss the potential influence of this issue.**

*Thank you for highlighting this. We have now updated figure 2 to display the combined G0 and G1 results, and the results stratified by G0 and G1, allowing readers to explore how the different data collection methods for G1 participants may have impacted participation between the generations. We have also now added additional text to the discussion regarding this:*

*“A further potential limitation is that whilst data collection for G0 participants was comparable to previous ALSPAC COVID-19 questionnaires (i.e., email invitation to an online-only questionnaire with no incentive), for G1 participants the methodology for the fourth COVID-19 questionnaire differed slightly* *as it was part of our standard annual questionnaire process. This meant that: 1) both email and postal invitations were sent; 2) approximately 1,000 paper questionnaires were received; and 3) there was a £10 gift voucher incentive. This resulted in a larger G1 sample size for this COVID-19 questionnaire, which may impact our results, both between the G0 and G1 generations and for G1 participation compared to previous COVID-19 questionnaires. First, the G1 sample with questionnaire data is substantially larger than for previous COVID-19 questionnaires (4,370 at the fourth questionnaire, vs 2,973 and 2,711 at the first and second questionnaires, respectively). While larger sample sizes are positive and potentially allows us to fill in missing information in earlier questionnaires, G1 participants who only completed the fourth questionnaire will not have previous COVID-19 data from earlier in the pandemic which may limit their use in longitudinal analyses assessing the progressive impact of the pandemic on participants. Second, focusing just on G1 participants who were previously sent a second COVID-19 questionnaire, response rates were higher than for previous questionnaires (65% response vs 51% and 44% response rates in the first and second questionnaires, respectively), suggesting that the incentive enhanced response (additional details can be found in the ‘response rates’ section). Together, this different data collection strategy may have altered factors associated with response among G1 participants, compared to previous data collections. Focusing just on the G1-specifc results in Figure 2 (in blue), G1 participants who only completed the fourth COVID-19 questionnaire, relative to those who completed the fourth COVID-19 questionnaire plus at least one previous questionnaire, were more likely to be male and have fewer educational qualifications. These differences were less pronounced in the G0-specific findings (red results in Figure 2), suggesting that they may have been due to the different data collection methods employed for G1 participants. However, whether these differences are solely due to the different data collection methods, as opposed to other differences between the generations, is difficult to infer from these results. Nonetheless, researchers should be aware of these different data collection techniques when using these data.”*

**Please provide references when comparing the response rates between different surveys. In addition, it was not quite clear what data were collected from the previous three surveys. Therefore, a table to summarize all variables or data in the four surveys might be helpful.**

*We have now added references to the response rates for the previous ALSPAC COVID-19 questionnaires in the ‘response rate’ section. Whilst we agree that a table summarising all of the variables in ALSPAC’s COVID-19 questionnaires may be helpful, as ALSPAC’s COVID-19 data collection is still on-going we believe that it is not necessary to add in here. The content of this fourth COVID-19 questionnaire is listed clearly in the ‘content design’ section, which includes details of which questions were repeated from previous COVID-19 questionnaires. Researchers interested in knowing which questions were asked the specific questionnaires can refer to the relevant data notes, or the ALSPAC data dictionary and variable search tool (described in the text).*

**I am not quite convinced to use the variable “predicted COVID-19 cases”, particularly if this was the outcome variable. Sensitivity and specificity of the variable should be provided.**

*We have now provided the sensitivity (0.65) and specificity (0.78) as reported in the original study in the main text. We are of course aware that this variable will contain measurement error, and in the discussion mention this and discuss work combining multiple sources of data together that is currently underway to try and improve case status estimation in ALSPAC (using self-reported case status, self-reported symptoms, serology tests and medical record linkage).*

**I am not sure whether the authors could use serological testing in the future to know the infection status, given that many of the participants could have obtained the vaccines. Should the vaccine information be included in the questionnaires?**

*At the time of the fourth COVID-19 questionnaire one serology test had already been conducted in October 2020 (Northstone et al., 2021; reference 9). Since then, a second serological test has been conducted and published, which we have now also referenced in the paper (Major-Smith et al., 2021; reference 23).*

*Regarding vaccine status, at the time this fourth questionnaire was conducted the vaccination campaign in the UK had only just begun, hence why it was not included. However, information regarding vaccinations has been asked about in the most recent COVID-19 questionnaire which is still being collected.*

**Perhaps the authors could consider adding the major challenges and a future perspective in the context of COVID-19 pandemic.**

*This is an interesting suggestion, and while we feel that this could be a worthwhile discussion topic, it is probably not appropriate for a data note which aims simply to describe the data. Discussion of ‘major challenges’ would be a better fit for a paper with a broader-perspective and we will absolutely bear this in mind as the data is used in more formal research.*

# Peer review: Reviewer response for version 1

**DOI:** 10.21956/wellcomeopenres.18704.r45565

**Published:** 2021-09-08

**Authors:** Holly Hope

**Affiliations:** 1 Centre for Women’s Mental Health, School of Health Sciences, University of Manchester, Oxford Rd, Manchester, UK

This report describes the fourth COVID survey issued to the ALSPAC cohort. The data note describes the survey, the COVID related information captured, and the survey respondents.

 Overall this is well written and the limitations of these data are addressed, particularly with reference to determination of COVID cases.

 My only comment would be that I found it confusing that this is the 4th COVID questionnaire. And that some respondents completed up to 3 previously in the past year.

 Based on the methods it appears that responses may overlap - and i wonder if there is a plan to derive variable across the four surveys.

 A sentence/table describing when the four COVID surveys were conducted and to what extent (if at all) they overlap, temporally, would be helpful.

Are sufficient details of methods and materials provided to allow replication by others?

Yes

Is the rationale for creating the dataset(s) clearly described?

Yes

Are the datasets clearly presented in a useable and accessible format?

Yes

Are the protocols appropriate and is the work technically sound?

Yes

Reviewer Expertise:

Epidemiology, behavioural science

I confirm that I have read this submission and believe that I have an appropriate level of expertise to confirm that it is of an acceptable scientific standard.

# Response

**Published:** 2021-11-26

**Authors:** Daniel Smith

**Affiliations:** University of Bristol, UK

We are delighted with the incredibly positive review and thank the reviewer for their constructive comments.

 The dates of the three previous questionnaires were given in the introduction, none of which overlapped: “So far, ALSPAC has conducted three COVID-19 questionnaires using G0 and G1 data: the first between 9th April to 15 ^th^ May 2020, the second between 26th May to 5th July 2020; and the third, with an antibody test, between 3rd and 20th October 2020.”

 In previous COVID-19 questionnaires, data collection for the G0 (the parental generation of mothers and partners/fathers) and G1 (the index study children) generations occurred at the same time, but for reasons described in the text for the fourth COVID-19 questionnaire the G0 data collection started prior to G1 data collection (and with G1 data collection continuing after G0 data collection ended). We assume that this is what the reviewer is referring to by ‘it appears that responses may overlap’?

 We have made this clearer in the updated manuscript in the ‘setting’ section of the methods by adding the following sentence: “Therefore, due to these differing data collection methods, unlike previous COVID-19 questionnaires where G0 and G1 data collection started and ended on the same dates, for this fourth COVID questionnaire start and end dates differed slightly between G0 and G1 participants (as detailed below in the 'Invitation and reminder strategy' section).”

 The ‘Invitation and reminder strategy’ section then goes on to say that G0 data collection for the fourth COVID-19 questionnaire started on 26 ^th^ November 2020 and ended on 7 ^th^ February 2021, while G1 data collection started on 2 ^nd^ December 2020 and ended on 19 ^th^ March 2021. We hope that this clarifies any confusion.

 Regarding variables derived across surveys, some variables have indeed been constructed and are described in the data note (Table 5). These derived variables relate to self-reported COVID-19 status and predicted COVID-19 cases based on an algorithm using self-reported symptoms; both questions have been asked in each of the four COVID-19 questionnaires.
